# The Effects of Body Position on Chemotherapy-Induced Nausea and Vomiting: A Single-Blind Randomized Controlled Trial

**DOI:** 10.5812/ircmj.17778

**Published:** 2014-06-05

**Authors:** Mohammad Fathi, Alireza Nikbakht Nasrabadi, Sina Valiee

**Affiliations:** 1 School of Nursing and Midwifery, Tehran University of Medical Sciences, Tehran, IR Iran; 2 School of Nursing and Midwifery, Kurdistan University of Medical Sciences, Sanandaj, IR Iran

**Keywords:** Nursing, Cancer, Nausea, Vomiting

## Abstract

**Background:**

Chemotherapy is the cornerstone of cancer treatment; however, alongside therapeutic effects, nausea and vomiting are two common complications of chemotherapy.

**Objectives:**

The aim of this study was to investigate the effects of body position on chemotherapy-induced nausea and vomiting.

**Materials and Methods:**

This was a single-blind randomized controlled clinical trial. We recruited a convenience sample of 79 patients and randomly allocated them to either experimental or control groups. Patients in the control group received chemotherapy in supine position while the experimental group received chemotherapy in semi-Fowler’s position. All patients were assessed for the severity, duration, and frequency of nausea and vomiting episodes every three hours up to 24 hours, ie, in nine time-points. Study data was analyzed by SPSS v. 16.

**Results:**

The severity, duration, and frequency of nausea and the severity and frequency of vomiting episodes in the control group differed significantly across the nine measurement time-points (P < 0.001). In the experimental group, the severity (P = 0.254) and frequency of nausea (P = 0.002) episodes as well as the frequency of vomiting (P = 0.008) episodes differed significantly across the measurement time-points. Moreover, the study groups differed significantly across the measurement time-point in terms of the severity (P < 0.001), duration (P < 0.001), and frequency of nausea (P = 0.002) and the severity (P < 0.001) and frequency (P < 0.001) of vomiting episodes.

**Conclusions:**

Compared to supine position, semi-Fowler’s position is more effective in relieving chemotherapy-induced nausea and vomiting.

## 1. Background

Cancer is a major health problem worldwide (1). It caused more than 7.6 million death in 2008, a mortality rate of about 13%. Most of these deaths happened in low-income and middle-income countries. It is estimated that by 2030, cancer-related deaths increase to 13 million worldwide (2). Moreover, according to the American Cancer Society (2013), the number of new cases of cancer in the United States in 2013 will be 1.6 million (3). In Iran, cancer is the third leading cause of death after cardiovascular diseases and accidents (4). Chemotherapy is the cornerstone of cancer treatment (5). As a systemic therapy, it destroys cancer cells even at remote parts of the body (6, 7). Chemotherapy, which is currently given to more than 50% of patients with cancer, saves millions of lives and brings many patients back to life (8). In the United States, about one million patients with cancer undergo chemotherapy yearly (9). Alongside therapeutic effects, chemotherapy exerts many side effects (10, 11). According to Roffe and Ernst, 80% of chemotherapies have some side effects. Due to its toxic effects, e.g. diarrhea, nausea, and vomiting, some patients consider chemotherapy unacceptable and unbearable (11). Sharma et al. reported that nausea and vomiting are respectively the first and the fourth most common side effects of chemotherapy (12). Firouzkuhi et al. also found that 54% to 96% of the patients receiving chemotherapy experienced nausea and vomiting (13, 14); however, 59% of adolescents participating in Baker and Ellett’s study reported that the side effects resulted more from cancer itself rather than from cancer treatments (15).

Side effects of chemotherapy progressively worsen patients’ condition and cause anxiety and depression and hence, can decrease patients’ compliance with treatment regimens. Uncontrolled nausea and vomiting delay the administration of chemotherapy and significantly reduce patients’ quality of life (16, 17). Hamadani et al. also found that 70% to 80% of the patients with cancer considered nausea and vomiting as the most debilitating side effects of chemotherapy. Besides, 46% to 50% of the patients who had participated in their study were thinking about the withdrawal of treatment (18). Many prevention and treatment strategies have been developed to manage chemotherapy-induced nausea and vomiting. For instance, antiemetic medications such as serotonin 5-HT3-receptor antagonists can significantly decrease the incidence of nausea and vomiting (19). Nonetheless, the incidence of chemotherapy-induced nausea and vomiting is still as high as 60% to 72% (20). Moreover, antiemetic agents have, in turn, many debilitating side effects such as headache, constipation, fatigue, mouth dryness, dizziness, diarrhea, drowsiness, akathisia, and extrapyramidal signs and symptoms (19), which can aggravate patients’ condition. Robertson et al. noted that there was no standard prophylactic treatment for preventing and managing chemotherapy-related complications (21). Consequently, nonpharmacological complementary and alternative therapies are currently administered, either alternatively or in combination with conventional treatments, for managing these complications (20). Nonpharmacological therapies prescribed for the management of chemotherapy-induced nausea and vomiting included, but not limited to, acupuncture, acupressure, relaxation, biofeedback, self-hypnosis, distraction, guided imagery, music therapy, and herbal medications such as Ginger (22, 23). An important nonpharmacological intervention for the management of chemotherapy-induced nausea and vomiting is patient positioning. Shahdadi et al. found that in comparison with supine position, semi-Fowler’s position significantly reduced the frequency, severity, and length of nausea in patients undergoing hemodialysis (24). Robertson et al. also found that compared with supine position, post-myelography nausea and vomiting was less frequent and less severe in semi-Fowler’s position (21). Rezaei-Adaryani et al. reported that positioning significantly decreased low back pain, improved physical comfort, and shortened the length of hospital stay after coronary angiography (25). Firouzkuhi et al. also reported that in comparison with supine position, patients receiving chemotherapy experienced fewer, shorter, and milder episodes of nausea and vomiting in semi-Fowler’s position (13, 14). Despite these findings, there is no integrated standard protocol for patient positioning during chemotherapy in our country, Iran, and patients received chemotherapy mainly in supine position.

## 2. Objectives

This study aimed to investigate the effects of body positioning on chemotherapy-induced nausea and vomiting.

## 3. Materials and Methods

**Figure 1.**
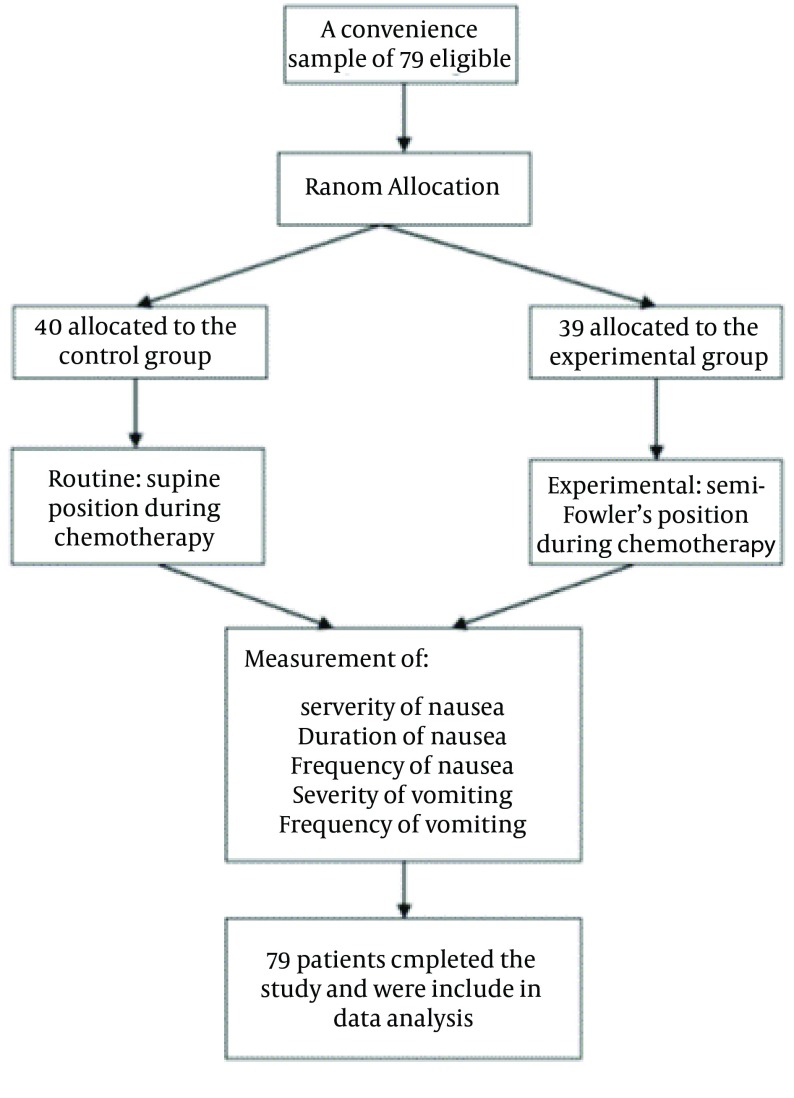
CONSORT Flowchart, Displaying the Flow of Subjects From Enrollment Through Analysis

This was a single-blind randomized controlled trial conducted in 2011. The study setting was the referral oncology ward of a 350-bed general teaching hospital affiliated with Kurdistan University of Medical Sciences, Sanandaj, Iran. The study population consisted of all patients with cancer referred to the study setting for receiving chemotherapy. The inclusion criteria were being older than 18 years of age, a definite diagnosis of any types of cancer, receiving single-day chemotherapy courses, and the history of receiving at least two courses of chemotherapy. The exclusion criteria were inability to assume or remain in the supine and semi-Fowler’s positions as well as need for emergency interventions during chemotherapy. Moreover, patients with electrolyte imbalance and pregnant women were excluded. Based on sample size formula, with delta of 1.4, an alpha of 0.05, and a power of 0.80, the sample size was determined as 31 patients in each group. Accordingly, we recruited a convenience sample of 79 patients for the study. Patients were randomly allocated to either experimental or control groups by using the permuted block randomization design. All participants were blind to the group assignment. Patients in the control group underwent no position change. Accordingly, they received chemotherapy in supine position. In the experimental group, patients received chemotherapy while they were placed in semi-Fowler’s position for three to four hours. All the patients in both groups received chemotherapy at a same time of day (from 8:00 to 14:00) and at a same environment. Moreover, the chemotherapy protocol and the nausea and vomiting treatment protocol were the same for both groups (Figure 1).

All patients were assessed for the severity, duration, and frequency of nausea episodes and the severity and frequency of vomiting episodes for 24 hours. The assessment time-points were immediately before chemotherapy and every three hours throughout and after chemotherapy. The study instrument consisted of a demographic questionnaire and a visual analogue scale (VAS) for assessing the severity of nausea and vomiting. The demographic and clinical data questionnaire was developed based on a literature review. Then, we invited ten nursing lecturers to assess the content validity of the questionnaire. The questionnaire was revised based on their comments. A standard VAS was used for evaluating the severity of nausea and vomiting episodes; in our study, VAS was a ten-centimeter ruler on which zero stood for no nausea or vomiting and ten stood for the most severe nausea and vomiting. VAS is routinely used worldwide for assessing the severity of nausea and vomiting (26-30). Wood et al. also noted VAS as a valid and reliable tool for assessing subjective measures (26). We also assessed the frequency of nausea and vomiting episodes by observing and interviewing patients. The duration of nausea and vomiting episodes was measured by using a chronometer. Study data was analyzed by using the SPSS v. 16.0 (SPSS Inc., Chicago, IL, USA). We assessed the normality of the study variables by using the Kolmogorov-Smirnov test. The result of this test revealed that all the study variables had a normal distribution. Accordingly, we employed parametric statistical tests for data analysis. We compared the study groups in terms of demographic variables by using the independent-samples t and the Chi square tests. Finally, we employed the repeated-measures analysis of variance (repeated-measure ANOVA) test to compare the severity, duration, and frequency of nausea and vomiting both within groups and between groups across the nine measurement time-points. All statistical assumptions related to the repeated measure ANOVA test were fulfilled. The level of significance was set at below 0.05. The Institutional Review Board and Ethics Committee of Kurdistan University of Medical Sciences approved the study. Moreover, the study was registered at the Iranian Registry of Clinical Trials (http://www.irct.ir/) with the registration number of IRCT201111268208N1. A written informed consent was obtained from all the study participants.

## 4. Results

Totally, 79 patients participated in this study: 40 in the experimental and 39 in the control groups. The mean patients’ age in the experimental and the control groups were 53.33 ± 15.61 and 56.67 ± 13.44, respectively. Most of the study participants (55.69%) were females. The most common type of cancer among the study participants was stage IV cancer of alimentary system. Finally, the most common therapeutic regimen administered to our participants consisted of mitotic inhibitor agents. The results of the independent-samples t and the Chi square tests revealed that the study groups did not differ significantly in terms of the demographic characteristics including age, gender, employment and educational status, and residency as well as clinical data including type and stage of cancer, length of disease, and treatment regimen (Tables 1 and 2)

**Table 1.** Demographic Characteristic of Control and Experimental Groups

	Control Group	Experimental Group	P Value
**Age, y ** ^**a**^	53.33 ± 15.61	56.67 ± 13.44	0.31^c^
**Gender ** ^**b**^			0.43^c^
Male	16 (40)	19 (48.7)	-
Female	24 (60)	20 (51.3)	-
**Educational Status** ^**b**^			0.09^d^
Illiterate	22 (55)	27 (69.2)	-
Elementary	11 (27.5)	3 (7.7)	-
Diploma	3 (7.5)	6 (15.4)	-
Higher	4 (10)	3 (7.7)	-
**Employment** ^**b**^			0.57^c^
Employed	23 (57.5)	20 (51.3)	-
Unemployed	17 (47.2)	19 (48.7)	-
**Residency** ^**b**^			0.75^c^
City	27 (67.5)	25 (64.1)	-
Rural	13 (32.5)	14 (35.9)	-

^a^ Data are presented as mean ± SD.

^c^ Chi square test.

^b^ Date are presented as No. (%).

^d^ Fisher’s exact test.

**Table 2.** Clinical Characteristic of Control and Experimental Groups ^a^

	Control Group	Experimental Group	P Value
**Type of Cancer**			0.67^b^
Respiratory	1 (0.02)	3 (0.08)	-
Gastrointestinal	19 (52)	15 (42)	-
Breast and Gynecology	3 (0.08)	2 (0.05)	-
Kidney	3 (0.08)	6 (0.17)	-
Skin	3 (0.08)	4 (0.11)	-
Others	7 (0.19)	5 (0.14)	-
**Stage of Cancer**			0.55^c^
Primary	12 (44)	11 (36)	-
Metastatic	15 (56)	19(64)	-
**Period of Chemotherapy**			0.46^c^
< 5	20 (55)	26 (68)	-
6-9	12 (33)	8 (21)	-
> 10	4 (11)	4 (11)	-
**Interval of Chemotherapy**			0.82^c^
1-3 week	14 (37)	12 (34)	-
3 <	24 (63)	23 (66)	-
**Treatment Regimen**			0.82^c^
Alkylating Agents	3 (0.07)	5 (0.13)	-
Antimetabolites	8 (0.2)	9 (0.23)	-
Mitotic Inhibitors	21 (0.53)	19 (0.49)	-
Topoisomerase Inhibitors	8 (0.2)	6 (0.15)	-

^a^ Data are Presented as No. (%).

^b^ Chi square test.

^c^ Fisher’s exact test.

On the other hand, the results of the repeated-measures ANOVA test for the within-subjects factor of time revealed that the severity, duration, and frequency of nausea and the severity and frequency of vomiting episodes in the control group differed significantly across the nine measurement time-points (P < 0.001; Table 3). The results of this test showed that in the experimental group, the severity (P = 0.003) and frequency (P = 0.002) of nausea episodes as well as the frequency of vomiting episodes (P = 0.008) differed significantly across the nine measurement time-points (Table 3). Moreover, the results of the repeated-measures ANOVA test for the between-subjects factor of group demonstrated that the study groups differed significantly across the nine measurement time-point in terms of the severity, duration, and frequency of nausea and the severity and frequency of vomiting episodes (Table 3). The trend of changes also revealed that in comparison with supine position, semi-Fowler’s position was more effective in reducing the severity, duration, and frequency of nausea and the severity and frequency of vomiting episodes (Table 3).

**Table 3.** Comparison of Dependent Variables Within-Subjects and Between-Subject of Control and Experimental Groups ^a^

	Severity of Nausea		Duration of Nausea		Frequency of Nausea		Severity of Vomiting		Frequency of Vomiting	
	Con.	Exp.	Con.	Exp.	Con.	Exp.	Con.	Exp.	Con.	Exp.
**Chemotherapy Start**	0.27 ± 0.81	0.18 ± 0.72	2 ± 3.55	1.23 ± 1.89	0.25 ± 0.63	0.69 ± 0.92	0.08 ± 0.35	0.1 ± 0.3	0.2 ± 0.68	0.26 ± 0.49
**3**	0.42 ± 0.98	0.46 ± 0.91	1.78 ± 3.36	0.97 ± 2.1	0.27 ± 0.71	0.31 ± 0.69	0.42 ± 0.98	0.44 ± 0.85	0.22 ± 0.42	0.18 ± 0.45
**6**	1.3 ± 1.92	0.77 ± 1.08	1.48 ± 3.87	0.59 ± 1.44	0.45 ± 0.87	0.15 ± 0.36	1.08 ± 1.55	0.33 ± 1.06	0.6 ± 1.21	0.15 ± 0.81
**9**	1.35 ± 1.99	0.33 ± 1.03	1.8 ± 3.96	0.31 ± 1.62	0.65 ± 0.94	0.08 ± 0.35	0.53 ± 1.17	0	0.25 ± 0.58	0
**12**	0.6 ± 1.42	0.8 ± 0.35	0.83 ± 2.41	0.05 ± 0.32	0.25 ± 0.58	0.03 ± 0.16	0.53 ± 1.46	0	0.1 ± 0.37	0
**15**	0.52 ± 1.84	0	0.4 ± 1.66	0	0.1 ± 0.37	0	0.13 ± 0.79	0	0.03 ± 0.15	0
**18**	0.13 ± 0.51	0.15 ± 0.96	0.2 ± 0.64	0.41 ± 2.56	0.1 ± 0.37	0	0.2 ± 0.72	0	0.03 ± 0.26	0
**21**	0.8 ± 0.47	0	0.8 ± 0.35	0	0	0	0.1 ± 0.63	0	0.8 ± 0.26	0
**24**	0.15 ± 0.58	0.1 ± 0.44	0.13 ± 0.56	0.23 ± 1.06	0.03 ± 0.15	0	0	0.08	0	0
**P Value within-subjects**	0	0.254	0	0.62	0	0.002	0	0.003	0	0.008
**P Value between-subjects**	0		0		0.002		0		0	

^a^ Abbreviations: Con., control group; and Exp., experiment group.

## 5. Discussion

The aim of this study was to investigate the effects of body positioning on chemotherapy-induced nausea and vomiting. Study findings revealed that in comparison with supine position, patients experienced less severe nausea in semi-Fowler’s position. Firouzkuhi et al. and Shahdadi et al. also found that in contrast to supine position, nausea episodes were less severe in semi-Fowler’s position (13, 14, 24). These findings confirm the effectiveness of semi-Fowler’s position in reducing the severity of chemotherapy-induced nausea. Moreover, the study findings demonstrated that in contrary to the patients in the control group, patients in the experimental group experienced shorter episodes of nausea. In addition, Firouzkuhi et al., Shahdadi et al. and Robertson et al. reported the same finding (13, 14, 21, 24). On the other hand, our study results showed that except for the T3 time-point, the trend of nausea frequency in the experimental group was downward. We also found that in comparison with the control group, nausea episodes were significantly less frequent in the experimental group. Shahdadi et al. also found that in contrast to the supine position, the number of hemodialysis-induced nausea episodes was significantly lower in semi-Fowler’s position (24). Firouzkuhi et al. also reported the same finding for chemotherapy-induced nausea (13). These findings support the effectiveness of semi-Fowler’s position in decreasing the frequency of chemotherapy-induced nausea.

Our study findings also revealed that the severity of vomiting episodes in the experimental group was significantly lower than the control group across all the nine measurement time-points. Firouzkuhi et al. also found that in comparison with supine position, vomiting episodes were less severe in semi-Fowler’s position (12). Moreover, our study findings demonstrated that the frequency of the vomiting episodes in the experimental group was significantly lower than the control group. Besides, from T4 to T8, patients in the experimental group experienced no episodes of vomiting. Firouzkuhi et al. also reported the same finding (11, 12). 

To summarize, in comparison with supine position, patients in semi-Fowler’s position generally experienced fewer, shorter, and milder episodes of nausea and vomiting. This can be attributed to many factors. In semi-Fowler’s position, gravity lifts abdominal organs and structures away from diaphragm and stomach. Consequently, intra-gastric pressure and diaphragmatic movements decrease, which in turn relieve nausea and vomiting. Study findings indicated that in contrast to the supine position, semi-Fowler’s position is more effective in reducing the severity, duration, and frequency of nausea and the severity and frequency of vomiting episodes in patients receiving chemotherapy. Given the simplicity, safety, and cost-effectiveness of semi-Fowler’s position, healthcare providers can relieve chemotherapy-induced nausea and vomiting by placing patients in this position.

### 5.1. Limitations and Recommendations

We selected a convenience sample for this study. Accordingly, the study findings might have limited generalizability. Moreover, in this study, we compared the effectiveness of only two positions in relieving chemotherapy-induced nausea and vomiting. Consequently, investigating the effects of other body positions on chemotherapy-induced nausea and vomiting is recommended. Investigating the effects of body positioning on nausea and vomiting induced by other clinical conditions is also recommended.
